# Plasma metabolomic profiling of hypertrophic cardiomyopathy patients before and after surgical myectomy suggests postoperative improvement in metabolic function

**DOI:** 10.1186/s12872-021-02437-0

**Published:** 2021-12-28

**Authors:** Nicole L. Wolter, Madison J. LeClair, Michael T. Chin

**Affiliations:** 1 Graduate School of Biomedical Sciences, Tufts University School of Medicine, Boston, USA; 2 Molecular Cardiology Research Institute, Tufts Medical Center, Boston, USA

**Keywords:** Hypertrophic cardiomyopathy, Metabolomics, Cardiovascular disease, Myectomy surgery

## Abstract

**Background:**

Hypertrophic cardiomyopathy (HCM) is a common inherited heart disorder complicated by left ventricle outflow tract (LVOT) obstruction, which can be treated with surgical myectomy. To date, no reliable biomarkers for LVOT obstruction exist. We hypothesized that metabolomic biomarkers for LVOT obstruction may be detectable in plasma from HCM patients.

**Methods:**

We conducted metabolomic profiling on plasma samples of 18 HCM patients before and after surgical myectomy, using a commercially available metabolomics platform.

**Results:**

We found that 215 metabolites were altered in the postoperative state (*p*-value < 0.05). 12 of these metabolites were notably significant after adjusting for multiple comparisons (*q*-value < 0.05), including bilirubin, PFOS, PFOA, 3,5-dichloro-2,6-dihydroxybenzoic acid, 2-hydroxylaurate, trigonelline and 6 unidentified compounds, which support improved organ metabolic function and increased lean soft tissue mass.

**Conclusions:**

These findings suggest improved organ metabolic function after surgical relief of LVOT obstruction in HCM and further underscore the beneficial systemic effects of surgical myectomy.

**Supplementary Information:**

The online version contains supplementary material available at 10.1186/s12872-021-02437-0.

## Background

Hypertrophic cardiomyopathy (HCM) is the most common inherited heart disorder [1]. HCM is characterized by hypertrophy of the left ventricle that is often asymmetrical and follows an autosomal dominant inheritance pattern. Patients with HCM experience complications including atrial fibrillation, sudden cardiac death, and left ventricular outflow tract (LVOT) obstruction [2]. Because LVOT obstruction is a significant cause of morbidity affecting 70% of HCM patients, but is often dynamic and not present without provocation [3], a biomarker for LVOT obstruction may be useful, particularly in situations where provocative testing with imaging is not available. The recommended treatment for patients with symptomatic LVOT obstruction is surgical myectomy, which shows high efficacy in reducing the detrimental effects of HCM. Alcohol septal ablation, an endovascular procedure, is recommended for those in whom surgical risk is deemed excessive, but sometimes gives incomplete relief of LVOT obstruction, thus providing another situation where a biomarker would prove useful. Although the relief of LVOT obstruction is of obvious hemodynamic benefit, little is known about the systemic and molecular changes that ensue. We have recently reported that surgical myectomy in HCM patients with LVOT obstruction is associated with changes in the plasma proteome consistent with reduction in systemic inflammation and improvement in physiological function, suggesting significant benefits beyond hemodynamic improvement [4]. To further explore this concept and to identify potential metabolite biomarkers, we investigated metabolomic changes between the pre- and post-surgical patient states.

Global metabolomic analysis has been significantly improved with advances in ultra-high performance liquid chromatography-tandem mass spectrometry (UPLC-MS/MS) technology and is currently being explored for its utility in providing clinically relevant information about various disease states including cardiovascular disease [5, 6]. Metabolomics analysis of pre- and postoperative states has already been conducted in various diseases, establishing the utility of investigating metabolomic alterations to aid clinical decision making [7–9]. Metabolomic analysis of HCM patient plasma samples before and after surgical myectomy could also aid in distinguishing the LVOT phenotype, as an alternative to currently used methods such as imaging or cardiopulmonary exercise testing, which may not always be accessible. Significant changes between the pre- and post-surgical metabolic states could also provide clinically useful information as an additional point of consideration when debating whether surgery may be the best option for a patient, as metabolomic profiles suggesting improved metabolic function may be a favorable outcome from surgery. Here, we measure the plasma metabolome of 18 HCM patients before and after surgical myectomy for LVOT obstruction. We show for the first time that there are metabolite changes in the postoperative state consistent with potential reduction in systemic inflammation, potential improvement in fatty acid metabolism, improvement in liver and kidney function, and an increase in lean soft tissue mass.

## Methods

### Study design

A total of 18 patients with clinically documented HCM referred and scheduled for surgical myectomy were approached for written informed consent to participate in the study and were included if they planned to follow up at our center. There were no other inclusion or exclusion criteria. Those who consented underwent a venous blood draw at their preoperative evaluation, within 4 weeks of their scheduled procedure. Follow up blood draws were performed at their HCM clinic postoperative visit, approximately 3 months after surgery, as was done in a prior study of plasma proteomics in this population [4]. The study was approved by the Tufts University/Medical Center Health Sciences Institutional Review Board under IRB protocol # 9487. All subjects gave their informed consent for inclusion before they participated in the study. The study was conducted in accordance with the Declaration of Helsinki. Patient characteristics were obtained from the medical record and are shown in Table 1.

**Table 1:** Patient summary demographics and clinical characteristics

Patient	2795	2799	2804	2815	2824	2829	2834	2841	2869	2855	2818	2875	2887	2916	5009	5038	5072	5119	Summary
Age at myectomy	37	43	55	56	73	73	58	63	52	76	63	73	65	62	35	41	55	54	Avg 57.4 ± 12.5
Female	Yes	No	Yes	Yes	Yes	Yes	No	No	Yes	Yes	Yes	Yes	No	Yes	No	No	Yes	Yes	67%
Prior AF	No	No	No	No	No	No	No	Yes	No	No	Yes	No	No	No	No	No	No	No	11%
Prior VT/VF	Yes	No	No	No	Yes	No	No	No	No	No	No	No	No	No	No	No	No	No	11%
Fam Hx SCD	Yes	No	No	No	No	No	No	No	No	No	No	No	No	No	No	No	No	No	6%
Fam Hx HCM	Yes	No	Yes	No	No	No	No	No	No	Yes	No	No	No	No	No	No	No	No	17%
*Medications*																			
Beta blocker	Yes	Yes	Yes	Yes	Yes	No	Yes	Yes	Yes	Yes	Yes	Yes	No	Yes	Yes	Yes	Yes	Yes	89%
Calcium channel blocker	No	No	No	Yes	No	Yes	No	No	No	No	Yes	No	No	Yes	No	No	No	No	22%
ACE or ARB	No	No	No	No	No	No	No	No	Yes	No	No	No	Yes	Yes	No	No	No	Yes	22%
Diuretic use	No	No	No	Yes	No	No	Yes	No	No	No	Yes	No	No	Yes	No	No	No	No	22%
*Physiological measurement*																			
Systolic blood pressure	135	128	110	170	105	126	142	126	148	128	122	117	140	130	130	140	128	118	Avg 130.2 ± 14.8
Diastolic blood pressure	80	82	80	70	56	78	90	78	74	78	70	70	80	70	90	70	75	70	Avg 75.6 ± 8.0
IVS thickness (mm)	23	13	15	15	17	15	15	18	14	23	17	18	17	16	15	21	17	12	Avg 16.7 ± 3.0
LVEF (%)	65	70	65	65	70	65	65	65	65	60	65	65	60	70	65	60	70	70	Avg 65.6 ± 3.4
LVOT gradient max (mm Hg)	60	60	85	90	100	150	110	100	145	150	100	100	80	64	160	100	90	100	Avg 102.4 ± 30.6
Pathogenic HCM Variant	MYBPC3	NF	NF	NF	MYBPC3	NF	NF	NF	NF	NF	NF	NF	NF	NF	NF	NF	MYH7	NF	17%

AF, atrial fibrillation; VT/VF, ventricular tachycardia or ventricular fibrillation; SCD, sudden cardiac death; HCM, hypertrophic cardiomyopathy; MR, mitral regurgitation; NF, not found; ND, not done

### Blood sample processing

Blood samples were collected in K2 EDTA tubes and centrifuged at 2000 g for 15 min at 4 °C to separate cells from plasma. The supernatant plasma was then aliquoted and stored at − 80 °C.

### Metabolomic profiling

Plasma samples gathered before and after surgical myectomy were sent for commercial metabolomic profiling (Metabolon, Morrisville, NC) using UPLC-MS/MS. The company uses a standardized sample preparation and analysis pipeline, as previously described [10]. Briefly, proteins were removed by methanol precipitation and centrifugation and split into 5 aliquots. Organic solvent was removed from the resulting extracts by brief placement on a TurboVAP (Zymark) and samples were then stored overnight under nitrogen prior to analysis. One aliquot was saved for backup while the other 4 were used for analysis. Aliquots were dried and then reconstituted in buffers compatible with subsequent UPLC-MS/MS. Two aliquots were analyzed using reverse-phase (RP)/UPLC-MS/MS with positive ion mode electrospray ionization (ESI), one was analyzed with RP/UPLC-MS/MS with negative ion mode ESI and one aliquot was analyzed by HILIC/UPLC-MS/MS with negative ion mode ESI. All methods utilized a Waters ACQUITY ultra-performance liquid chromatography (UPLC) and a Thermo Scientific Q-Exactive high resolution/accurate mass spectrometer interfaced with a heated electrospray ionization (HESI-II) source and Orbitrap mass analyzer operated at 35,000 mass resolution. Each reconstitution solvent contained a series of standards at fixed concentrations to ensure injection and chromatographic consistency. One aliquot was analyzed using acidic positive ion conditions, chromatographically optimized for more hydrophilic compounds. In this method, the extract was gradient eluted from a C18 column (Waters UPLC BEH C18-2.1 × 100 mm, 1.7 µm) using water and methanol, containing 0.05% perfluoropentanoic acid (PFPA) and 0.1% formic acid (FA). Another aliquot was also analyzed using acidic positive ion conditions; however it was chromatographically optimized for more hydrophobic compounds. In this method, the extract was gradient eluted from the same afore mentioned C18 column using methanol, acetonitrile, water, 0.05% PFPA and 0.01% FA and was operated at an overall higher organic content. Another aliquot was analyzed using basic negative ion optimized conditions using a separate dedicated C18 column. The basic extracts were gradient eluted from the column using methanol and water, however with 6.5 mM Ammonium Bicarbonate at pH 8. The fourth aliquot was analyzed via negative ionization following elution from a HILIC column (Waters UPLC BEH Amide 2.1 × 150 mm, 1.7 µm) using a gradient consisting of water and acetonitrile with 10 mM Ammonium Formate, pH 10.8. The MS analysis alternated between MS and data-dependent MS^n^ scans using dynamic exclusion. The scan range varied slighted between methods but covered 70–1000 m/z. Raw data was extracted, peak-identified and QC processed using Metabolon’s hardware and software. Compounds were identified by comparison to library entries of purified standards or recurrent unknown entities. Metabolon maintains a library based on authenticated standards that contains the retention time/index (RI), mass to charge ratio (*m/z)*, and chromatographic data (including MS/MS spectral data) on all molecules present in the library. Furthermore, biochemical identifications are based on three criteria: retention index within a narrow RI window of the proposed identification, accurate mass match to the library ± 10 ppm, and the MS/MS forward and reverse scores between the experimental data and authentic standards. The MS/MS scores are based on a comparison of the ions present in the experimental spectrum to the ions present in the library spectrum. While there may be similarities between these molecules based on one of these factors, the use of all three data points can be utilized to distinguish and differentiate biochemicals.

### Statistical analysis

Matched pairs *t-*tests were performed on log transformed paired patient samples pre- and post-myectomy to obtain an initial list of potentially important metabolites. The matched pairs t-test is equivalent to the one-sample t-test performed on the differences of the observations taken on each subject (i.e., calculate (x_1_ – x_2_) for each subject; test whether the mean difference is zero or not). The test statistic is given by ${\text{t}} = { }\left( {{\overline{\text{x}}}_{1} - {\overline{\text{x}}}_{2} } \right)/{\text{n}}$, with n – 1 degrees of freedom, where ${\overline{\text{x}}}_{1}$, ${\overline{\text{x}}}_{2}$ are the sample means for groups 1 and 2, respectively, s_d_ is the standard deviation of the differences, n is the number of subjects (so there are 2n observations). A volcano plot was generated using RStudio, with BioConductor package EnhancedVolcano (https://github.com/kevinblighe/EnhancedVolcano). Hierarchical clustering was performed on log transformed data using ArrayStudio (Qiagen Digital Insights, Redwood City, CA).

The false discovery rate (FDR) was also calculated to account for multiple comparisons between patients. The FDR for a given set of compounds can be estimated using the *q*-value. In order to interpret the *q*-value, the data must first be sorted by the *p*-value then choose the cutoff for significance (typically *p* < 0.05). The *q*-value gives the false discovery rate for the selected list (i.e., an estimate of the proportion of false discoveries for the list of compounds whose *p*-value is below the cutoff for significance) [11].

## Results

### Patient cohort characteristics

18 patients were enrolled into this pilot study. Patient characteristics are summarized in Table 1 and listed more extensively in Additional file 1: Table S1. HCM patients referred for surgical myectomy at Tufts Medical Center were approached consecutively and those that gave consent and planned to return for a postoperative visit were enrolled. The patients varied in age from 37 to 76. Twelve of eighteen were female (67%) and fifteen of eighteen had NYHA heart failure classification of 3 or greater (83%). Two of the patients carried pathogenic Mybpc3 mutations, one carried a pathogenic Myh7 mutation, and 15 patients had no pathogenic mutations found during screening (83%). Two out of eighteen patients had a history of atrial fibrillation (11%) and two of eighteen had a history of ventricular tachycardia or ventricular fibrillation leading to ICD placement (11%). Seventeen of eighteen had medical comorbidities in addition to HCM. Sixteen out of eighteen were taking beta blockers. LVOT gradients were documented for all patients, either at rest or with provocation, ranging from 60 to 160 mm Hg, with a mean maximal gradient of 102.4 ± 30.6. Thirteen of eighteen (72%) had at least mild mitral regurgitation. No patients had midventricular obstruction. All 18 patients underwent surgical myectomy, while five had concurrent mitral valve surgery, two had concurrent coronary artery bypass grafting and two had aortic valve replacement for concurrent aortic stenosis. The two patients with atrial fibrillation had concurrent MAZE procedures. All had no residual LVOT gradient on follow up echocardiogram done around the time of the postoperative visit.

### Metabolomic profiling demonstrates within person stability of distinct metabolite fingerprints

**Fig. 1:**
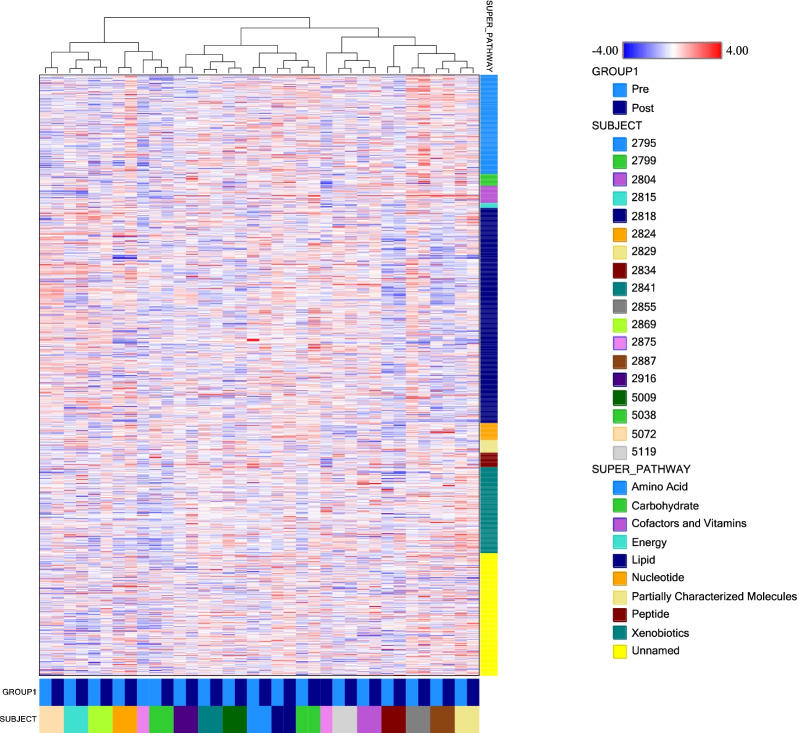
Hierarchical clustering of plasma metabolomic profiles sorts by patient identity

Metabolomic analysis was performed on paired plasma samples from 18 patients. This identified a total of 1,340 metabolites. We wanted to first understand in more detail the proteome profiles of these samples and the relationships of the individual pre- and post-surgery samples based on relative concentration of all 1,340 metabolites identified. Consequently, we performed hierarchical clustering using all samples across all metabolites (Fig. 1). Hierarchical clustering sorts samples by similarity of metabolite concentrations in an unbiased fashion and has the potential to reveal dominant biomarkers that could distinguish the preoperative, LVOT obstruction state from the postoperative state where LVOT obstruction is no longer present. Samples with a more comparable expression pattern cluster together and separate from samples with a more dissimilar expression pattern. This hierarchical cluster analysis of all samples with all metabolites demonstrated that each paired Pre/Post patient sample clustered together and separated from all other patients, with one exception, subject 2875 (Fig. 1). This patient had concurrent aortic stenosis and also underwent aortic valve replacement, and thus may be expected to have a more divergent shift in metabolomic profile. This result indicates that, in general, the overall expression profile of all metabolites is more closely related within a patient than between Pre- and Post-surgery, suggesting that each person has a unique overall plasma metabolite fingerprint distinct from any other person, and is consistent with previous plasma proteomic profiling in HCM patients before and after surgical myectomy [4]. No dominant metabolic biomarkers of the postoperative state that would drive clustering into the preoperative and postoperative states were found.

### Metabolomic profiling reveals an altered metabolome post-myectomy indicative of alterations in specific metabolic pathways

**Fig. 2:**
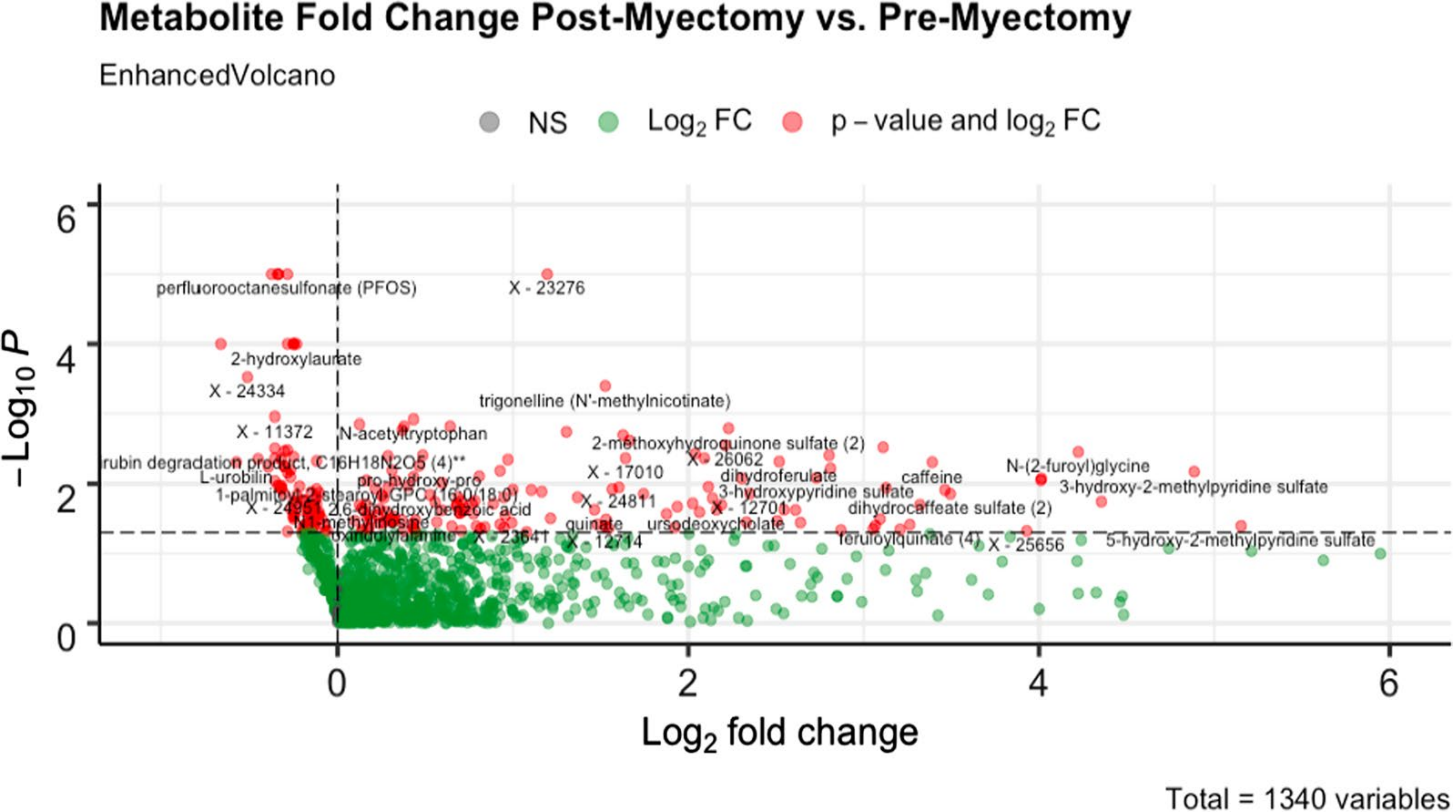
Volcano plot showing the fold-change of metabolites post-myectomy. Threshold for significance is *p*-value < 0.05

In order to measure metabolomic changes in post-operative patient plasma, metabolomic profiling was conducted. Briefly, patient plasma samples obtained before and after surgical myectomy were sent to Metabolon to be analyzed by mass spectrometry. Plasma metabolite levels were then compared by fold-change between the pre- and postoperative states for each patient. 1070 of these biochemicals were successfully identified while 270 remain structurally unidentified. All identified metabolites could be assigned to specific metabolic pathways and subpathways as listed in Additional file 2: Table S2. Of these, 215 metabolites exhibited a statistically significant fold-change post-myectomy, with a *p*-value < 0.05 by matched pairs t-test. These metabolites were further broken down into 139 metabolites upregulated and 76 metabolites downregulated post-myectomy, as shown in Fig. 2.

**Fig. 3:**
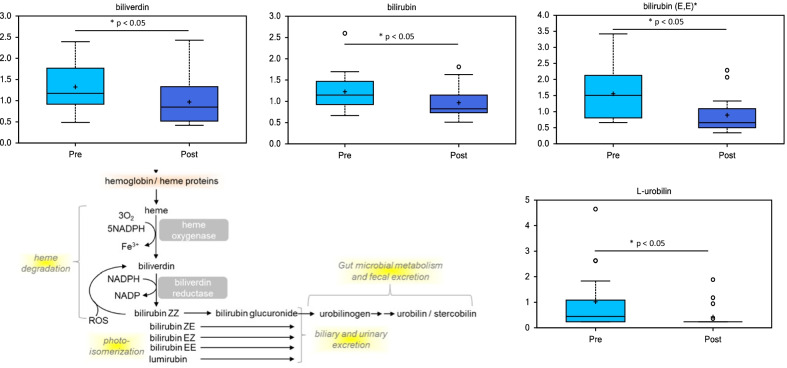
Hemoglobin metabolites such as biliverdin and bilirubin are decreased in HCM patient plasma after surgical myectomy. Metabolic pathway diagrams are derived from the KEGG database [12–14]

Review of the various metabolite changes across multiple metabolic pathways (Additional file 1: Table S1, Additional file 2: Table S2) revealed notable changes in metabolites for pathways such as Heme metabolism (Fig. 3). Heme metabolites include biliverdin and the bilirubins (Z,Z; E,E; E,Z or Z,E), which were significantly decreased. In addition, L-urobilin was decreased following surgery. When heme is broken down, it is first converted into biliverdin and bilirubin (via heme oxygenase (HO) and biliverdin reductase (BR) activities). After transport to the liver and excretion into the bile, bilirubin can be converted into urobilinogen via the gut microbiota. Subsequently, urobilinogen can be converted to either d-urobilin or l-urobilin.

**Fig. 4:**
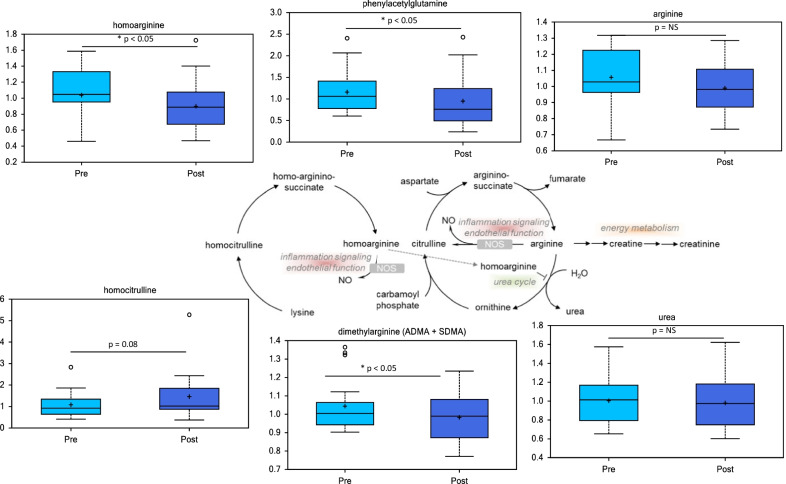
Arginine metabolites and a urea cycle derivative, phenylacetylglutamine, are altered in HCM patient plasma after surgical myectomy. Metabolic pathway diagrams are derived from the KEGG database [12–14]

Arginine metabolites were also altered after myectomy surgery, suggesting alterations in arginine metabolism and the urea cycle (Fig. 4). Homoarginine was significantly decreased while homocitrulline trended higher following surgery (Fig. 4). In addition, dimethylarginine (ADMA + SDMA) was also decreased following myectomy. Phenylacetylglutamine, an acetylated peptide that is a biomarker of urea cycle disorders, was also decreased following surgery.

**Fig. 5:**
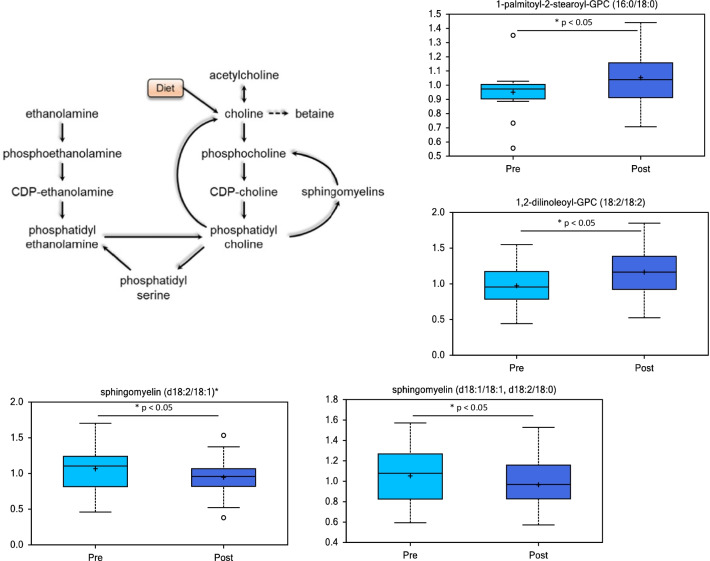
Phospholipid metabolites are altered in HCM patient plasma after surgical myectomy. Metabolic pathway diagrams are derived from the KEGG database [12–14]

Plasma phospholipids and sphingomyelins also showed a variety of changes after myectomy surgery (Fig. 5). Phospholipids are synthesized from diacylglycerols and polar head groups such as choline or ethanolamine and circulate in plasma as constituents of lipoproteins synthesized mostly in the liver. Phosphatidylcholine (PC) is the major phospholipid found in lipoproteins and is required for lipoprotein assembly and secretion. PCs (e.g. 1-palmitoyl-2-stearoyl-GPC (16:0/18:0) and 1,2-dilinoleoyl-GPC (18:2/18:2)), some phosphatidylinositol (PI) species and several phosphatidylethanolamine (PE) species were increased following surgery (Fig. 5 and Additional file 1: Table S1, Additional file 2: Table S2). There were also decreases in plasma for many sphingomyelins (e.g. sphingomyelin (d18:1/18:1, d18:2/18:0) and sphingomyelin (d18:2/18:1)) following myectomy. Sphingomyelins are phospholipids derivatives lacking glycerol backbones; they are composed of ceramide (a lipid made up of sphingosine and a fatty acid) and a polar head group (phosphocholine in most cases). Sphingomyelins are present in high levels in lipoproteins and these data suggest that the composition of lipoproteins change following myectomy.

**Table 2:** Significantly downregulated and upregulated metabolites in the postoperative state

Metabolite	Fold change	*P* value	*Q* value
Perfluorooctanesulfonate (PFOS)	0.82	5.74E−08	4.52E−05
Perfluorooctanoate (PFOA)	0.77	9.69E−08	4.52E−05
3,5-dichloro-2,6-dihydroxybenzoic acid	0.79	5.22E−07	2.00E−04
2-hydroxylaurate	0.85	6.58E−05	8.80E−03
Bilirubin (E,E)	0.63	1.00E−04	1.17E−02
Trigonelline (N′-methylnicotinate)	2.88	4.00E−04	3.14E−02
X-21339	0.79	1.61E−06	3.00E−04
X-23276	2.29	2.03E−06	4.00E−04
X-16935	0.84	1.00E−04	8.80E−03
X-11308	0.82	1.00E−04	1.17E−02
X-17654	0.82	1.00E−04	1.17E−02
X-24334	0.7	3.00E−04	2.61E−02

**Fig. 6:**
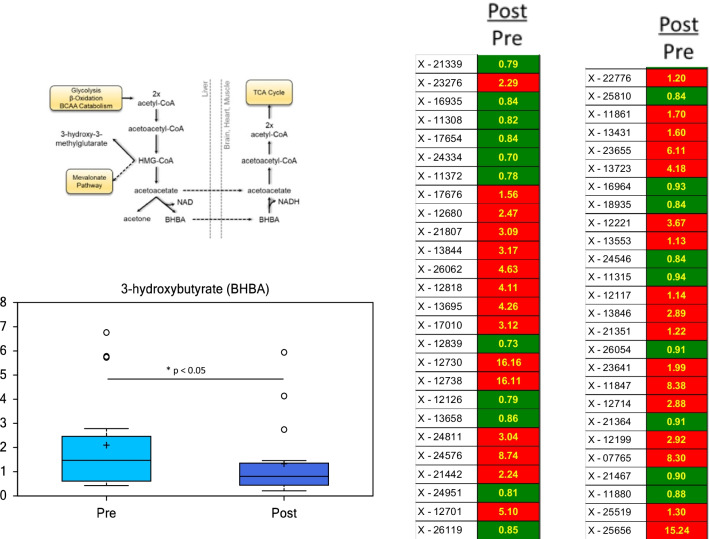
BHBA and a variety of unknown compounds are altered in HCM patient plasma after surgical myectomy

Another metabolite that showed a significant change was 3-hydroxybutyrate (BHBA), which was decreased following myectomy (Fig. 6). BHBA (a ketone body) is produced in the liver, secreted and used by other tissues, including the heart where it can be converted to acetyl-CoA to support energetic needs. Biochemicals associated with food intake, xanthine/caffeine and benzoate metabolites (which are also the products of metabolism of plant polyphenols) were increased following surgery. This may reflect an increase in food intake as the patients return to normal following surgery and/or reflect improved circulation in the patients. Unnamed compounds are discrete biochemicals which do not correspond to a standard in the Metabolon library. Of the 270 unnamed compounds that were identified, 52 were significantly different (*p* ≤ 0.05) following myectomy.

### High stringency screening for altered metabolites reveals additional potential metabolic pathways altered after surgical myectomy

Although many metabolites show significant alterations in paired t-testing, the relatively small number of patients and the multiple metabolites being compared cannot exclude type 1 error. To account for multiple statistical comparisons between metabolites, the false discovery rate (*q*-value) was also calculated for each metabolite. When applying a more stringent significance threshold of *q*-value < 0.05, 12 metabolites were identified to meet this threshold (Table 2). Six of these metabolites were structurally identified. These were perfluorooctanesulfonate (PFOS), perfluorooctanoate (PFOA), 3,5-dichloro-2,6-dihydroxybenzoic acid, 2-hydroxylaurate and bilirubin, which were all downregulated with fold-changes of 0.82, 0.77, 0.79, 0.85, and 0.63, respectively, and trigonelline, which was upregulated with a fold-change of 2.88. Five metabolites that were unable to be structurally unidentified were significantly downregulated in the postoperative state, while one was upregulated.

## Discussion

By analyzing the plasma metabolome of HCM patients before and after surgical myectomy, we have demonstrated, for the first time, that the plasma metabolomic profiles of HCM patients exhibit measurable, important changes in the postoperative state reflective of improvement in organ metabolic function. Although patient metabolic profiles sorted more by patient identity than by operative state, as is usually the case in patient studies of this nature, and we did not find dominant biomarkers that clearly separated the preoperative from the postoperative state in all patients, we were still able to identify broad shifts in metabolite patterns that inform potential metabolic changes in HCM patients after myectomy surgery. Specifically, we were able to identify trends in bilirubin, arginine derivatives, phospholipids and other metabolites that suggest important physiological changes as outlined below.

Bilirubin, biliverdin and urobilin are products of heme metabolism via HO and BR enzymes, which concludes with the conjugation of unconjugated bilirubin in hepatocytes. Increased serum levels of bilirubin are generally associated with decreased overall liver function, and bilirubin is a well-established clinical biomarker for liver function [15]. In our study, plasma bilirubin showed a fold-change of 0.63 (*p*-value < 0.0001, *q*-value < 0.05) following surgical myectomy, which demonstrates that plasma levels of bilirubin are decreased in the postoperative state. These data therefore may suggest decreased HO or BR enzyme activities but may also indicate an increase in biliary and urinary excretion, potentially indicating improved liver and kidney function. Another indication of increased kidney function may be the decrease in a group of acetylated peptides, specifically phenylacetylglutamine, a biomarker of urea cycle disorders that is normally excreted via urine [16].

Homoarginine and dimethylarginine are components of the urea cycle, important for removal of nitrogenous waste product. Homoarginine can mediate inhibition of arginase activity and inflammation [17]. In addition, homoarginine may support nitric oxide (NO) synthesis by serving as a substrate for nitric oxide synthase (NOS) and by inhibiting arginase activity [18]. Reduced homoarginine would be consistent with a possible decrease in NO production and inflammation following surgery. Consistent with this finding, we note that none of our 18 patients had increased vasopressor requirements in the postoperative period. We did not do any further studies to assess the relationship between blood pressure and arginine metabolites in our patients. Dimethylarginine has been linked to increased risk for heart failure in human studies [19] and reduction of this metabolite is consistent with improved cardiac function. Taken together, these data are consistent with improvement in markers of cardiac health and inflammation.

The observed increases in circulating phospholipid metabolites may reflect improved secretion of lipoproteins from the liver. Importantly, previous studies suggest that circulating levels of PC are lower in people with heart damage compared to healthy adults [20], thus the increases in the current study may reflect improved heart function. Cardiomyopathy can also result in the heart relying increasingly more on glucose utilization than on fatty acid oxidation for energy, hence a decrease in plasma BHBA may reflect an improvement in fatty acid usage by the heart. The concurrent reduction in circulating sphingolipid metabolites is also interesting, as circulating sphingolipids, especially ceramides, have been postulated to promote type 2 diabetes via pathways involved in insulin resistance, pancreatic islet β-cell dysfunction and inflammation [21]. Patients with HCM who undergo surgery thus may also demonstrate improved endocrine function.

PFOS and PFOA are man-made chemicals that function as fluoro-polymers and are used for industrial purposes. These chemicals have both been found to have potentially adverse effects on liver function, and have been associated with increased levels of hepatocellular injury biomarkers in humans [22] as well as increased risk of chronic kidney disease [23]. Here, we show that plasma levels of PFOA and PFOS decrease following surgical myectomy in HCM patients, with fold-changes of 0.77 (*p*-value < 0.0000001, *q*-value < 0.00005) and 0.82 (*p*-value < 0.0000001, *q*-value < 0.00005), respectively. Decreased plasma levels of these chemicals are consistent with improved liver and kidney function resulting in more effective clearance of these chemicals from the systemic circulation. HCM is known to confer risk of end stage renal disease [24], and myectomy may reduce this risk. HCM may impact the liver and kidney function of patients by inducing congestive hepatopathy and reduced renal blood flow at a subclinical level, as has been described for other cardiovascular diseases such as left heart failure, cardiomyopathy, and constrictive pericardial disease [25], and this impact is likely mitigated after surgical myectomy.

3,5-dichloro-2,6-dihydroxybenzoic acid has been reported as a biomarker for red meat and dairy intake [26]. Our study showed that 3,5-dichloro-2,6-dihydroxybenzoic acid was decreased in plasma post-myectomy with a fold-change of 0.79 (*p*-value < 0.000001, *q*-value < 0.0005). We speculate that reduction in plasma 3,5-dichloro-2,6-dihydroxybenzoic acid is in the postoperative state is indicative of improved liver and kidney function resulting in more rapid clearance, although a change in dietary intake of red meat and dairy products cannot be ruled out.

2-hydroxylaurate is a lipid that is mainly important as chemical modification of lipopolysaccharide (LPS) and is specifically added to the endotoxic portion Lipid A used by all gram-negative bacteria as a virulence factor [27]. LPS is cleared by the liver through portal circulation [28], suggesting a possible mechanism by which 2-hydroxylaurate appears in human plasma. Though studies of LPS clearance by the liver have not yet elucidated its metabolic mechanism, we propose that 2-hydroxylaurate may be a product of LPS breakdown. We demonstrate that 2-hydroxylaurate is decreased in plasma post-myectomy with a fold-change of 0.85 (*p*-value < 0.0001, *q*-value < 0.01). As with PFOS and PFOA, it is possible that 2-hydroxylaurate is decreased in the postoperative state because the liver is more efficiently clearing this metabolite.

Trigonelline (N-methylnicotinic acid) is a product of niacin metabolism and is found most prominently within coffee seeds, among other foods [29]. Our study showed that trigonelline levels showed a fold change of 2.88 (*p*-value = 0.0004, *q*-value < 0.05) in the postoperative state, being the only identified metabolite to show a statistically significant increase in plasma level (Table 2). Trigonelline is also associated with lean soft tissue mass in cancer patients, which correlates with muscle mass [30]. It is possible that the postoperative state is associated with improved lean soft tissue mass as a result of greater functional capacity, although a confounding factor such as recent coffee intake cannot be ruled out. Studies of trigonelline bioavailability have shown that maximum plasma levels of trigonelline occur around 2–3 h after coffee ingestion, with a half-life of approximately 5 h before being cleared in the urine [31, 32].

Curiously, five metabolites that were significantly downregulated in the postoperative state were unable to be structurally identified (Table 2). It is possible that, as with 2,3-dichloro-2,6-dihydroxybenzoic acid, there is little known about these metabolites because they may be byproducts of chemicals that have not yet been deeply investigated. Given that the other decreased metabolites that were successfully identified are cleared by the liver and/or kidney, we speculate that these metabolites could be other chemicals that are cleared by the liver and/or kidney. It will be necessary to further confirm the identity of these metabolites to validate this speculation and confirm their utility as biomarkers.

The overarching theme of our study is that metabolomic profiling suggests improvement in the metabolic function of various organs in HCM patients after myectomy to alleviate LVOT obstruction. These organs include the liver, kidney, heart, endocrine pancreas, cardiac and skeletal muscle. Although HCM has been associated with kidney and cardiac dysfunction [3, 24], associations with liver, pancreatic and skeletal muscle dysfunction in patients have not been described, although crosstalk between the liver and heart has been suggested in mouse models of HCM [33]. A limitation of our study is that many confounding factors related to nutritional status, such as Body Mass Index, diet, herbal remedies, tobacco use, etc. can contribute to metabolomic profiles and were not assessed in our patients but were noted in the above discussion where relevant. It is also important to note that established biomarkers for kidney function (creatinine), liver function (AST and ALT), inflammation (C-reactive protein) and heart failure (Brain Natriuretic Peptide) were not different in HCM patients in the preoperative and postoperative states (Additional file 1: Table S1, Additional file 2: Table S2, line 210; [4]). These findings argue that the observed changes in metabolites are not simply related to improvement in heart failure, renal perfusion, liver perfusion or inflammatory state but may be unique to HCM patients. A corollary speculation could be that these metabolites may be more sensitive to low levels of heart failure, reduced organ perfusion and inflammation in HCM patients than established markers, but this hypothesis requires further testing and validation. Potential clinical relationships between HCM with LVOT obstruction and liver dysfunction, islet cell dysfunction or skeletal muscle dysfunction in patients remain to be explored in future studies.

Our study is a pilot study limited by small sample size, patient heterogeneity and the need for larger scale validation and mechanistic studies in animal and/or in vitro models. The patient heterogeneity results from selection solely based on need for a surgical myectomy and plan to follow up postoperatively at our institution, without further inclusion or exclusion criteria. Our study population is skewed towards female patients (67%), shows significant age heterogeneity and has a low prevalence of sarcomere mutations (17%), as a result. A future, larger study will try to balance sex, age and sarcomere mutation status to avoid bias and to allow stratification based on these characteristics. Some patients had concurrent coronary artery disease, atrial fibrillation and one even had aortic stenosis, necessitating additional surgical intervention beyond myectomy such as coronary artery bypass grafting, MAZE procedures and in one case an aortic valve replacement. These additional conditions and the additional surgical procedures may confound our analysis. A future study with narrowed selection criteria limiting the condition to uncomplicated HCM with LVOT obstruction may uncover additional metabolites and pathways. Another limitation of our study is that many confounding factors related to nutritional status, such as Body Mass Index, diet, herbal remedies, tobacco use, etc. can contribute to metabolomic profiles. Information about these confounding factors was not assessed in our patients but was noted in the above discussion where relevant.

## Conclusions

Our work is the first, to the best of our knowledge, to analyze plasma metabolomes of HCM patients with LVOT obstruction before and after surgical myectomy. We demonstrate that the plasma metabolome is altered postoperatively, and that metabolites associated with cardiac, renal, liver and endocrine dysfunction are decreased in the postoperative state, while those associated with improvement in liver function and lean soft tissue mass are increased in the postoperative state. These findings suggest a metabolic benefit of surgical myectomy that goes beyond simple hemodynamic relief of LVOT obstruction and will inform future clinical and preclinical validation and mechanistic studies.

## Supplementary Information

**Additional file 1: Supplementary Table 1.** Detailed Patient Characteristics.

**Additional file 2:** Complete Metabolomic Profile of All Samples Tested.

## Data Availability

The entire metabolomics dataset is available as an online supplement to this article.

## Abbreviations

HCM: Hypertrophic cardiomyopathy
LVOT: Left ventricular outflow tract
BHBA: 3-Hydroxybutyrate
PFOS: Perfluorooctanesulfonate
PFOA: Perfluorooctanoate
UPLC-MS/MS: Ultra-high performance liquid chromatography-tandem mass spectrometry
ICD: Implantable cardioverter-defibrillator
HO: Heme oxygenase
BR: Biliverdin reductase
ADMA: Asymmetric dimethylarginine
SDMA: Symmetric dimethylarginine
PC: Phosphatidylcholine
PI: Phosphatidylinositol
PE: Phosphatidylethanolamine
NO: Nitric oxide
NOS: Nitric oxide synthase
LPS: Lipopolysaccharide
K2EDTA: Dipotassium ethylenediaminetetraacetic acid
ESI: Electrospray ionization
RP/UPLC-MS/MS: Reverse phase ultra-high performance liquid chromatography-tandem mass spectrometry
HESI: Heated electrospray ionization
PFPA: Perfluoropentanoic acid
FA: Formic acid
RI: Retention time index
FDR: False discovery rate

## References

[1] Semsarian C, Ingles J, Maron MS, Maron BJ (2015). New perspectives on the prevalence of hypertrophic cardiomyopathy. J Am Coll Cardiol.

[2] Maron BJ, Maron MS (2013). Hypertrophic cardiomyopathy. Lancet.

[3] Maron BJ, Longo DL (2018). Clinical course and management of hypertrophic cardiomyopathy. N Engl J Med.

[4] Larson A, Libermann TA, Bowditch H, Das G, Diakos N, Huggins GS (2021). Plasma proteomic profiling in hypertrophic cardiomyopathy patients before and after surgical myectomy reveals post-procedural reduction in systemic inflammation. Int J Mol Sci.

[5] Shimada YJ, Batra J, Kochav SM, Patel P, Jung J, Maurer MS (2021). Difference in metabolomic response to exercise between patients with and without hypertrophic cardiomyopathy. J Cardiovasc Transl Res.

[6] Ussher JR, Elmariah S, Gerszten RE, Dyck JR (2016). The emerging role of metabolomics in the diagnosis and prognosis of cardiovascular disease. J Am Coll Cardiol.

[7] Khan TA, Loftus TJ, Filiberto AC, Ozrazgat-Baslanti T, Ruppert MM, Bandyopadhyay S (2021). Metabolomic profiling for diagnosis and prognostication in surgery: a scoping review. Ann Surg.

[8] Liesenfeld DB, Habermann N, Toth R, Owen RW, Frei E, Staffa J (2015). Changes in urinary metabolic profiles of colorectal cancer patients enrolled in a prospective cohort study (ColoCare). Metabolomics.

[9] Samczuk P, Ciborowski M, Kretowski A (2018). Application of metabolomics to study effects of bariatric surgery. J Diabetes Res.

[10] Michealraj KA, Kumar SA, Kim LJY, Cavalli FMG, Przelicki D, Wojcik JB (2020). Metabolic regulation of the epigenome drives lethal infantile ependymoma. Cell.

[11] Storey JD, Tibshirani R (2003). Statistical significance for genomewide studies. Proc Natl Acad Sci U S A.

[12] Kanehisa M (2019). Toward understanding the origin and evolution of cellular organisms. Protein Sci.

[13] Kanehisa M, Furumichi M, Sato Y, Ishiguro-Watanabe M, Tanabe M (2021). KEGG: integrating viruses and cellular organisms. Nucleic Acids Res.

[14] Kanehisa M, Goto S (2000). KEGG: kyoto encyclopedia of genes and genomes. Nucleic Acids Res.

[15] Sullivan JI, Rockey DC (2017). Diagnosis and evaluation of hyperbilirubinemia. Curr Opin Gastroenterol.

[16] Mokhtarani M, Diaz GA, Rhead W, Lichter-Konecki U, Bartley J, Feigenbaum A (2012). Urinary phenylacetylglutamine as dosing biomarker for patients with urea cycle disorders. Mol Genet Metab.

[17] Marz W, Meinitzer A, Drechsler C, Pilz S, Krane V, Kleber ME (2010). Homoarginine, cardiovascular risk, and mortality. Circulation.

[18] Tommasi S, Elliot DJ, Da Boit M, Gray SR, Lewis BC, Mangoni AA (2018). Homoarginine and inhibition of human arginase activity: kinetic characterization and biological relevance. Sci Rep.

[19] Visser M, Paulus WJ, Vermeulen MA, Richir MC, Davids M, Wisselink W (2010). The role of asymmetric dimethylarginine and arginine in the failing heart and its vasculature. Eur J Heart Fail.

[20] Paapstel K, Kals J, Eha J, Tootsi K, Ottas A, Piir A (2018). Inverse relations of serum phosphatidylcholines and lysophosphatidylcholines with vascular damage and heart rate in patients with atherosclerosis. Nutr Metab Cardiovasc Dis.

[21] Yun H, Sun L, Wu Q, Zong G, Qi Q, Li H (2020). Associations among circulating sphingolipids, beta-cell function, and risk of developing type 2 diabetes: a population-based cohort study in China. PLoS Med.

[22] Gallo V, Leonardi G, Genser B, Lopez-Espinosa MJ, Frisbee SJ, Karlsson L (2012). Serum perfluorooctanoate (PFOA) and perfluorooctane sulfonate (PFOS) concentrations and liver function biomarkers in a population with elevated PFOA exposure. Environ Health Perspect.

[23] Shankar A, Xiao J, Ducatman A (2011). Perfluoroalkyl chemicals and chronic kidney disease in US adults. Am J Epidemiol.

[24] Lee H, Han K, Park JB, Hwang IC, Yoon YE, Park HE (2019). Risk of end-stage renal disease in patients with hypertrophic cardiomyopathy: a nationwide population-based cohort study. Sci Rep.

[25] El Hadi H, Di Vincenzo A, Vettor R, Rossato M (2020). Relationship between heart disease and liver disease: a two-way street. Cells.

[26] Wang Y, Hodge RA, Stevens VL, Hartman TJ, McCullough ML (2020). Identification and reproducibility of plasma metabolomic biomarkers of habitual food intake in a US diet validation study. Metabolites.

[27] Hittle LE, Powell DA, Jones JW, Tofigh M, Goodlett DR, Moskowitz SM (2015). Site-specific activity of the acyltransferases HtrB1 and HtrB2 in *Pseudomonas aeruginosa* lipid A biosynthesis. Pathog Dis.

[28] Su GL (2002). Lipopolysaccharides in liver injury: molecular mechanisms of Kupffer cell activation. Am J Physiol Gastrointest Liver Physiol.

[29] Ashihara H, Ludwig I, Katahira R, Yokota T, Fujimura T, Crozier A (2014). Trigonelline and related nicotinic acid metabolites: occurrence, biosynthesis, taxonomic considerations, and their roles in planta and in human health. Phytochem Rev.

[30] Stretch C, Eastman T, Mandal R, Eisner R, Wishart DS, Mourtzakis M (2012). Prediction of skeletal muscle and fat mass in patients with advanced cancer using a metabolomic approach. J Nutr.

[31] Lang R, Dieminger N, Beusch A, Lee YM, Dunkel A, Suess B (2013). Bioappearance and pharmacokinetics of bioactives upon coffee consumption. Anal Bioanal Chem.

[32] Lang R, Wahl A, Skurk T, Yagar EF, Schmiech L, Eggers R (2010). Development of a hydrophilic liquid interaction chromatography-high-performance liquid chromatography-tandem mass spectrometry based stable isotope dilution analysis and pharmacokinetic studies on bioactive pyridines in human plasma and urine after coffee consumption. Anal Chem.

[33] Magida JA, Leinwand LA (2014). Metabolic crosstalk between the heart and liver impacts familial hypertrophic cardiomyopathy. EMBO Mol Med.

